# Single-cell sequencing reveals the existence of fetal vascular endothelial stem cell-like cells in mouse liver

**DOI:** 10.1186/s13287-023-03460-y

**Published:** 2023-08-30

**Authors:** Fitriana N. Rahmawati, Tomohiro Iba, Hisamichi Naito, Shota Shimizu, Hirotaka Konishi, Weizhen Jia, Nobuyuki Takakura

**Affiliations:** 1 Department of Signal Transduction, Research Institute for Microbial Diseases, Osaka University, Suita, Osaka Japan; 2 Department of Physiology, School of Medicine, Kanazawa University, Kanazawa, Ishikawa Japan; 3 Immunology Frontier Research Center, Osaka University, Suita, Japan; 4 Center for Infectious Disease Education and Research, Osaka University, Suita, Japan; 5 Integrated Frontier Research for Medical Science Division, Institute for Open and Transdisciplinary Research Initiatives (OTRI), Osaka University, Suita, Japan

**Keywords:** Single-cell transcriptomic sequencing, Endothelial stem cells, Heterogeneity, Mouse liver, Trajectory analysis, RNA velocity, Development

## Abstract

**Background:**

A resident vascular endothelial stem cell (VESC) population expressing CD157 and CD200 has been identified recently in the adult mouse. However, the origin of this population and how it develops has not been characterized, nor has it been determined whether VESC-like cells are present during the perinatal period. Here, we investigated the presence of perinatal VESC-like cells and their relationship with the adult VESC-like cell population.

**Methods:**

We applied single-cell RNA sequencing of endothelial cells (ECs) from embryonic day (E) 14, E18, postnatal day (P) 7, P14, and week (W) 8 liver and investigated transcriptomic changes during liver EC development. We performed flow cytometry, immunofluorescence, colony formation assays, and transplantation assays to validate the presence of and to assess the function of CD157^+^ and CD200^+^ ECs in the perinatal period.

**Results:**

We identified CD200^−^ expressing VESC-like cells in the perinatal period. These cells formed colonies in vitro and had high proliferative ability. The RNA velocity tool and transplantation assay results indicated that the projected fate of this population was toward adult VESC-like cells expressing CD157 and CD200 1 week after birth.

**Conclusion:**

Our study provides a comprehensive atlas of liver EC development and documents VESC-like cell lineage commitment at single-cell resolution.

**Supplementary Information:**

The online version contains supplementary material available at 10.1186/s13287-023-03460-y.

## Introduction

Vascular development occurs by two distinct mechanisms: vasculogenesis and angiogenesis. Vasculogenesis was considered to be a process exclusively occurring during the embryonic period. However, it has also been suggested that this process can be mediated in the postnatal period by endothelial progenitor cells (EPCs) located in the bone marrow [1]. Since then, differences in endothelial cell (EC) properties reflected by an immature phenotype or high proliferative capacity have been sought in order to understand the contribution of these cells to blood vessel generation in the adult. In particular, identifying surface markers for adult ECs with stem cell potential would be useful for investigating the hierarchy of EC development and for tracing EC lineages. We recently identified a subset of CD157^+^CD200^+^-expressing resident ECs with the ability to proliferate and regenerate blood vessels in response to tissue injury [2]. According to the study, in adult mouse liver, these CD157^+^C200^+^ ECs mainly reside in the portal vein but are not present in the liver sinusoidal EC population. However, the existence, origin, and development of vascular endothelial stem cell (VESC)-like cells during fetal and neonatal periods are still obscure.

Vascularization of the mouse liver primordium begins as early as embryonic day (E) 8.5 when ECs of the unknown source are detected between the septum transversum mesenchyme (STM) and hepatic diverticulum. The liver vascular network is also formed by the contribution of preexisting vessels, namely, the vitelline, umbilical, and posterior cardinal veins [3]. It has been suggested that vascular ECs and sinusoids are derived from hemangioblasts in the early liver, although the endoderm and endocardium may also contribute to sinusoid development [4, 5]. In contrast with the sophisticated analysis of differentiation from hematopoietic stem cells (HSCs) to hematopoietic cells, the hierarchy of development from VESC-like cells to mature ECs remains unclear. Here, using single-cell RNA sequencing (scRNA-seq) of ECs from the fetal, neonatal, and adult periods, we provide evidence that adult CD157^+^C200^+^ VESCs emerge from fetal CD157^−^CD200^+^ VESC-like cells around the 1st week after birth. We document the heterogeneity of liver ECs during development and reveal a developmental hierarchy of VESC-like populations toward mature ECs through progenitor stages, using in silico methods.

## Materials and methods

### Animals

C57BL/6J and C57BL/6 Tg (CAG-EGFP) mice were purchased from Japan SLC (Shizuoka, Japan) and maintained under specific pathogen-free (SPF) condition under 12-h light–dark cycle in environmentally controlled rooms in an animal experimentation facility at Osaka University. For mouse embryos, the morning on which the vaginal plug was detected was defined as E0.5. All animal protocols and experiments were carried out in accordance with the guidelines of Osaka University Committee for animal and recombinant DNA experiments. Euthanasia was carried out by cervical dislocation for adult mice and decapitation with sharp surgical scissors for fetuses and neonates up to 10 days.

### Cell preparation and flow cytometry

ECs defined as CD31-positive, CD45-negative cells were isolated from liver (± 6 mice for each experiment) and sorted according to previously published protocols [6]. For fetal samples, incubation with shaking step was omitted. Cell surface staining was performed using the following antibodies: rat anti-mouse CD31 BV421 (Biolegend, San Diego, USA), rat anti-mouse CD45 FITC (eBiosciences, San Diego, USA), rat anti-mouse CD45 APC-Cy7 (Biolegend), mouse anti-mouse CD157 APC (Biolegend), and rat anti-mouse CD200 PE (Biolegend), all at 1:200 dilution. To exclude dead cells, propidium iodide (PI; Sigma-Aldrich Japan) was added before analysis and sorting on a BD FACSAria (BD Biosciences, San Diego, USA). Flow cytometry data quantification was performed using FlowJo v10 (BD Biosciences).

### Endothelial cell colony-forming assay

About 1 × 10^3^ primary ECs isolated as described above were seeded into 24-well plates and co-cultured with OP9 cells (RIKEN Cell Bank, Tsukuba, Japan). Cultures were maintained in RPMI 1640 medium (Sigma-Aldrich Japan, Tokyo, Japan) supplemented with 10% fetal bovine serum (FBS; Sigma-Aldrich Japan), 1% penicillin/streptomycin (Sigma-Aldrich Japan), and 10^–5^ mol/L 2-mercaptoethanol (Thermo Fisher Scientific K.K., Yokohama, Japan) with the addition of vascular endothelial growth factor (VEGF) at 10 ng/mL (PeproTech, Rocky Hill, USA) every 3 days. After 10 days, cells were fixed for immunostaining. Also, 1 × 10^4^ primary ECs were cultured in 35-mm glass-based dishes (Iwaki, Shizuoka, Japan) coated with laminin 511 at 1–2 μg/cm^2^ (Matrixome, Suita, Japan). HuMedia-EG2 (Kurabo, Osaka, Japan) supplemented with 10% FBS (Sigma-Aldrich Japan) with the addition of VEGF 10 ng/mL (PeproTech) was used to maintain the cultures for 10 days.

### Limiting dilution assay

Isolated primary ECs from CAG-EGFP mice were seeded into 96-well plates in twofold descending serial dilutions ranging from 1000 cells/well to 1 cell/well and co-cultured with OP9 cells. Cells were cultured for 10 days, and number of positive wells (wells containing colonies with at least 50 cells) counted. The number of positive wells and total wells tested was entered into an internet-based limiting dilution analysis software program from Walter and Eliza Hall Bioinformatics (http://bioinf.wehi.edu.au/software/elda/) [7].

### Immunohistochemistry and immunofluorescence staining

Cultured ECs were washed with PBS before fixation with 4% PFA in PBS for 15 min at room temperature (RT) followed by blocking buffer (PBST containing 5% NGS/1% BSA/0.2% skim milk) for 2 h at 4 °C. Cells were stained with unconjugated rat anti-mouse CD31 (Biolegend) at 4 °C overnight followed by biotin-conjugated polyclonal goat anti-rat IgG (Agilent Technologies, Santa Clara, USA) for 2 h and ABC reagent (Vector Laboratories, CA, USA) for 30 min at RT.

Dissected mouse liver tissues were fixed in 4% paraformaldehyde (PFA) at 4 °C for at least 8 h and incubated in 15% and 30% sucrose until cells sank. Fixed specimens were embedded in Tissue Tek Optimum Cutting Temperature (Sakura Finetek, Tokyo, Japan) compound and cut into 30-μm (fetal samples) or 50-μm (neonatal and adult samples) sections. After fixation with 4% PFA for 15 min and blocking buffer sections were incubated with unconjugated rat anti-mouse CD31 (Biolegend), unconjugated hamster anti-mouse CD31 (Merck Millipore, Darmstadt, Germany), mouse anti-mouse CD157 PE (Biolegend), rat anti-mouse CD200 (Biolegend), rat anti-mouse E-cadherin APC, or polyclonal rabbit anti-glutamine synthetase (Abcam, Cambridge, USA) antibodies at 4 °C overnight. After washing, sections were treated with goat anti-rat Alexa Fluor 488 IgG (Thermo Fisher), goat anti-hamster IgG Alexa Fluor 488 (Thermo Fisher), or goat anti-rabbit IgG Alexa Fluor 647 (Thermo Fisher). Slides were mounted using Dako Fluorescent Mounting Medium (Dako, California, USA).

Images were acquired using a Canon EOS Kiss×7 (Canon, Tokyo, Japan), Leica DMi8 and Leica TCS SP5 confocal microscope (Leica Microsystems, Nussloch, Germany) and processed with Leica application suite (Leica Microsystems), Image J (﻿National Institute of Health), QuPath [8], and Adobe Photoshop software (Adobe Systems, San Jose, USA).

### Single-cell RNA-seq library preparation

Fluorescence-activated cell sorting** (**FACS)-purified liver ECs were processed using the Chromium Single-Cell 3’ Kit v3.1 (10X Genomics, Pleasanton, CA) in accordance with the standard protocol. Resulting cDNAs and libraries were assessed using Agilent Bioanalyzer High Sensitivity Chips (Agilent Technologies) and sequenced on a DNBSEQ-G400 (Japan MGI Tech Co., Ltd., Japan). Aligning the sequencing reads to the mm10 mouse genome reference, and generating count tables of unique molecular identifiers (UMIs) was done using Cell Ranger v3.1.0 (10X Genomics).

### Single-cell RNA-seq analysis

Analysis was carried out using the R package Seurat v4.0.3 [9]. We excluded cells with fewer than 200 or more than 8000 distinct detected genes and more than 15% mitochondrial genes. Then, we performed log normalization scaled to 10.000 UMIs per cell. The Seurat Find Variable Genes function was applied to define highly variable genes as an input for principal component analysis. We performed normalization using SCTransform function and regressing out total count per cell, percentage of mitochondrial genes, and difference between S and G2M phase scores. FindNeighbors, FindClusters, and RunUMAP function using 30 principal components were used to determine the clusters. For combined analysis with published perinatal data [10], integration using harmony was performed to remove batch effect [11]. Cell annotations were ascribed by comparing differential expression for each cluster and matching to canonical cell-type markers from the literature. Trajectory analysis was performed using Monocle 3 [12–14]. For RNA velocity analysis, BAM files for each dataset were processed using the velocyto command line tool to generate Loom files containing spliced/unspliced transcript counts [15]. Velocities were calculated in Python 3.8.0 using scVelo visualized in the uniform manifold approximation and projection (UMAP) embedding obtained from Seurat analysis [16].

### GO term analysis

Differentially expressed genes (logfc.threshold 1, *p* < 0.001) were uploaded to the Metascape [17] website to analyze gene ontology (GO) term enrichment in each cluster.

### Transplantation model

Liver vascular injury was induced by intraperitoneal injection of monocrotaline (MCT) (Sigma-Aldrich Japan) at a dose of 500 mg/kg. Two days after, whole-body irradiation was performed with a single dose of 600 cGy. Eight hours after irradiation, 1 × 10^4^—5 × 10^4^ of sorted ECs from E18 EGFP mice were resuspended in 30-μL PBS (4%FBS) and transplanted into the spleen parenchyma using 27-gauge needle.

### Quantification and statistical analysis

All statistical analysis and graphing were performed using GraphPad Prism v9.0 (GraphPad Software, Inc., CA, US). Numerical data were tested for normality using Shapiro–Wilk normality tests followed by unpaired Student’s *t*-test. A *p* value of < 0.05 was considered statistically significant.

## Results

### Single-cell analysis of liver ECs across developmental stages

**Fig. 1:**
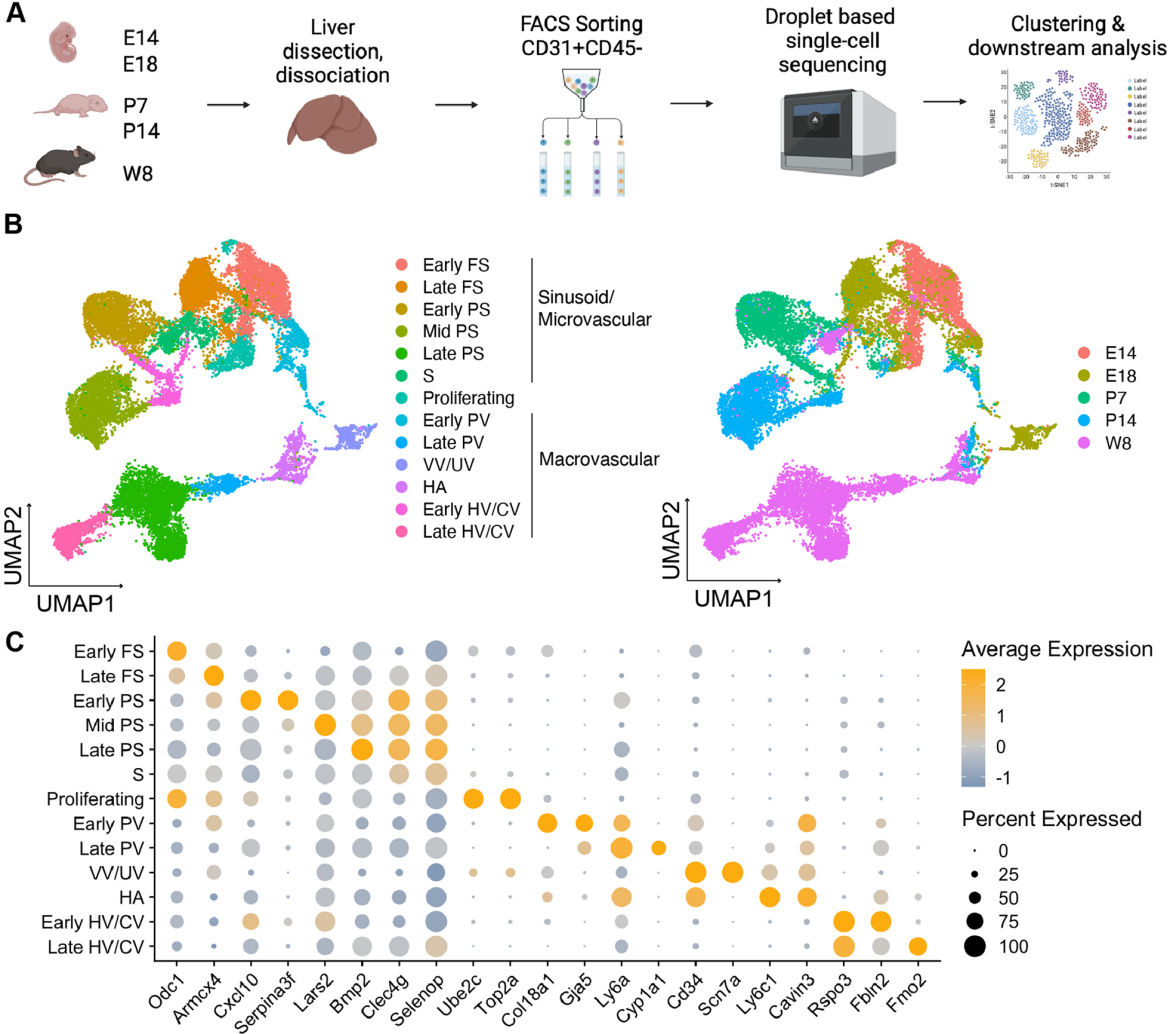
Single-cell analysis of liver ECs across development. **a** Schematic of experimental design for the single-cell analysis pipeline. Livers from E14, E18, P7, P14, and W8 were dissected and dissociated into single cells. Sorted CD31^+^CD45^−^ ECs were collected and analyzed using the 10X Genomics Chromium platform. For each time point, one scRNA-seq library was generated using pooled tissues dissected from ± 6 individual animals. **b** UMAP visualization of ECs from all time points (n = 20.799 cells) colored and labeled based on cell clusters (left) and time point (right). **c** Dot plot of differentially expressed genes in each cluster. Gene expression in Log10 (TPM)

To assess the EC heterogeneity during the development of liver ECs, we conducted a droplet-based single-cell analysis of female C57BL6/J mouse liver ECs collected at five different time points during development. This covered fetal (E14 and E18), neonatal (P7 and P14), and adult (W8) periods. CD31^+^CD45^−^ ECs from the dissected liver were sorted and profiled them using the 10X Genomics Chromium platform (Fig. 1a). After filtering out low-quality cells, liver cells clusters were visualized through UMAP (Additional file 1: Fig. S1A and S1B). EC clusters were selected based on the expression of *Pecam1*/*CD31* and *Cdh5*/*VE-cadherin* and contaminating lymphatic ECs (*Mmrn1*), bile duct (*Col3a1*), myeloid (*Pf4*), RBC (*Hba-a2*), kupffer cells (*Tyrobp*), and hepatocytes (*Apoa1*) were removed (Additional file 1: Fig. S1C). After re-clustering, 13 subpopulations within ECs including six sinusoid clusters, six macrovascular EC clusters, and a proliferating EC cluster were identified (Fig. 1b). The EC clusters and their gene expression patterns identified in this study were consistent with the previous single-cell analyses of liver ECs [10, 18–21]. Integration of our data with perinatal liver ECs data from Gómez-Salinero et al. [10] showed overlaps between the two datasets (Additional file 1: Fig. S1D). We observed a high degree of heterogeneity of sinusoids over time, represented by different clusters for each time point. The only exception was the S cluster, which was detected at all time points and had no specific marker expression (Fig. 1b and c). Within the sinusoidal group, we can divide the developmental time point into early fetal sinusoid (FS) (*Odc1*), late FS (*Armcx4*), early postnatal sinusoids (PS) (*Serpina3f*), mid-PS (*Lars2*), and late PS (*Bmp2*). This heterogeneity revealed a dramatic shift in EC gene expression between the early and late stages of development with the downregulation of the fetal genes *Mest* and *Fabp5,* which play essential roles in vascular development [22, 23], and the upregulation of *Cdkn1a* and *Fcgr2b,* which are involved in cell proliferation and senescence [24] (Additional file 1: Fig. S1E).

In contrast with earlier findings [10, 25], we found that heterogeneity was also present within macrovascular ECs. Portal vein (PV) ECs can be divided into early PV (*Col18a1*) from the fetal, early, and mid-postnatal period; and late PV (*Cyp1a1*), mainly from the late postnatal period (Fig. 1b and c, Additional file 1: Fig. S1F). With a similar distribution over developmental stages, hepatic vein (HV) and central vein (CV) ECs can be divided into early HV/CV (*Fbln2*) and late HV/CV (*Fmo2*). Notably, we found a separate cluster of vitelline vein/umbilical vein (VV/UV) (*Scn7a*) and hepatic artery (HA) (*Ly6c1*), which were previously described by Gómez-Salinero et al. as a single cluster expressing *Cavin3* [10]. This gene was also expressed in the PV cluster, confirming the contribution of VV/UV ECs to PV EC development [26] (Fig. 1c). The VV/UV EC cluster was formed exclusively by ECs from the fetal period and switched into the HA EC cluster during the late fetal period, which increases in frequency after birth (Additional file 1: Fig. S1F). Taken together, these data illustrate the heterogeneity and transcriptional changes of liver ECs during development.

### CD157^+^CD200^+^ ECs emerge from CD157^−^CD200^+^ ECs during the perinatal period

**Fig. 2:**
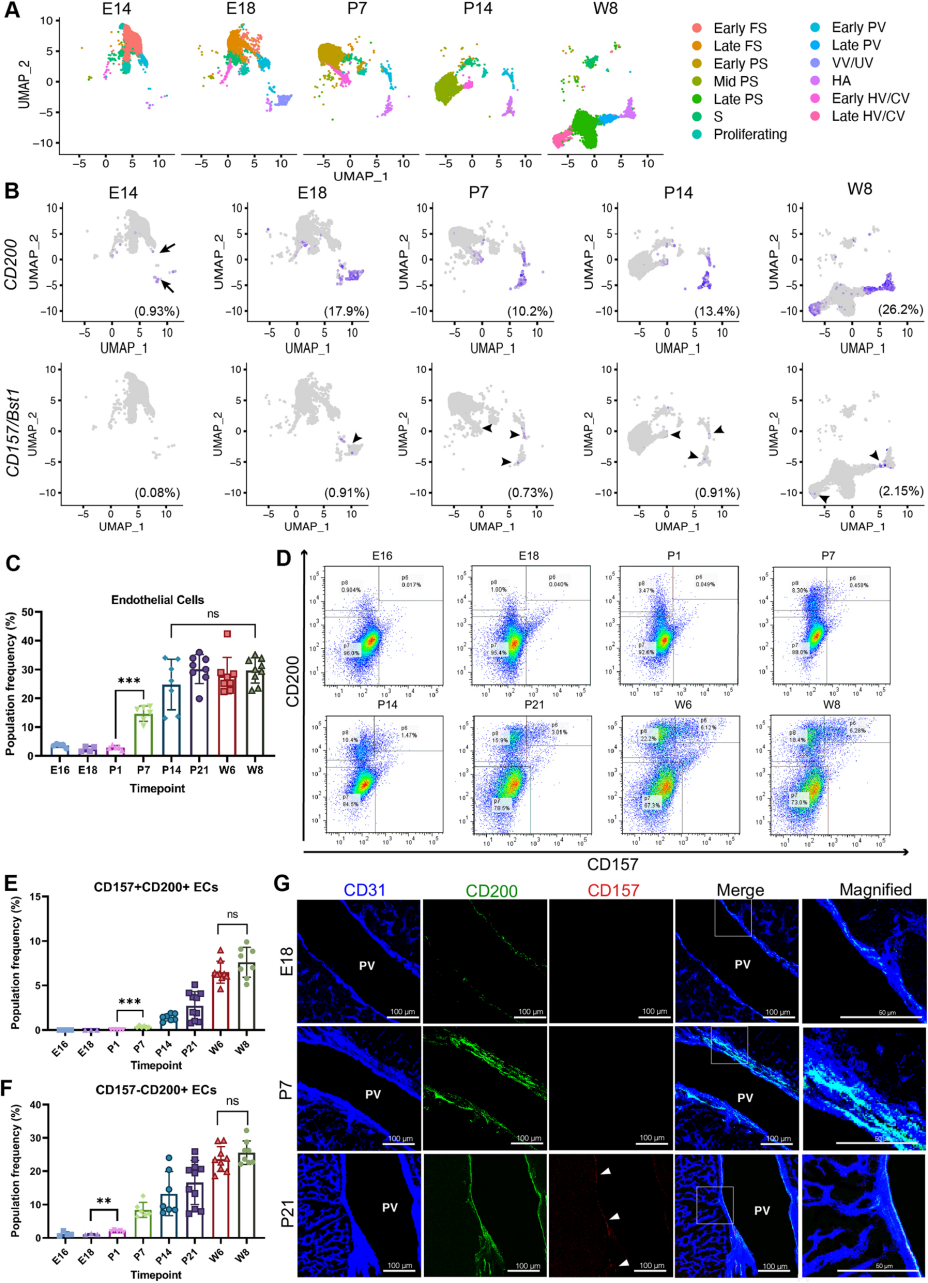
CD157^+^CD200^+^ VESCs emerge from CD157^−^CD200^+^ ECs during the perinatal period. **a** UMAP projection of each EC clusters from E14 to W8. **b** Feature plot showing relative distribution and expression of *CD157/Bst1* (top) and *CD200* (bottom) for each time point. Each purple dot represents a single cell. Black arrows: *CD200* + ECs; black arrowheads: *CD157* + ECs. **c** Quantification of total ECs among all cells in liver. **d** FACS analysis of the liver from different time points showing the emergence of CD157^+^CD200^+^ among CD31^+^CD45^−^ ECs during early postnatal period. **e, f** Quantification of CD157^+^CD200^+^ ECs **(e)** and CD157^−^CD200^+^ ECs **(f)** among total ECs. **g **Immunofluorescence analysis of CD157 and CD200 in portal vein of E18, P7, and P21 liver. White arrowheads show the expression of CD157 within the PV. PV, portal vein. Data are shown as means ± SEM. Statistical analysis using unpaired two-tailed t-test, *** *p* < 0.0001 and **p < 0.005, ns *p* > 0.05

To explore the emergence of adult VESCs or CD157^+^CD200^+^ ECs, we assessed the distribution of *CD157/Bst1* and *CD200* in EC clusters from all tested time points. *CD200* expression was detected as early as E14, mainly in PV, VV/UV, and HA EC clusters, and at E18 for the HV/CV EC cluster. Similarly, *CD157/Bst1* began to be expressed mainly in the VV/UV EC and less commonly in PV clusters at E18, then in HA and HV/CV clusters at P7 (Fig. 2a and b). ECs expressing both *CD157/Bst1* and *CD200* (CD157^+^CD200^+^ ECs) were first observed at E18. The percentage of CD157^+^CD200^+^ ECs slightly decreased slightly after birth due to the obliteration of VV/UV ECs but then increased again until adulthood (Additional file 1: Fig. S2A). To validate this result, we analyzed CD157^+^ and CD200^+^ ECs in the perinatal period by fluorescence-activated cell sorting (FACS) and immunofluorescence (IF). By using FACS, first CD31^+^CD45^−^-gated ECs were analyzed for the presence of CD157^+^CD200^+^ ECs populations (Additional file 1: S2B). The percentage of ECs among all liver cells during the fetal period was relatively low but increased dramatically during the first 2 weeks after birth (Fig. 2c). We found a low number of CD157^+^CD200^+^ ECs emerged around P7 and then gradually increased with age (Fig. 2d and e). Notably, the emergence and increase in CD157^+^CD200^+^ ECs were preceded by CD157^−^CD200^+^ ECs, and an age-dependent increase was similarly observed for both populations (Fig. 2d and f).

Next, CD157^+^ and CD200^+^ cells in the perinatal period were analyzed by IF staining. To differentiate between portal vein (PV) and central vein (CV) areas, we compared the expressions of CD157 and CD200 with E-cadherin and glutamine synthetase, markers for periportal and pericentral hepatocytes, respectively (Additional file 1: Fig. S2C and D) [27]. In adults, CD157^+^ ECs were abundant in the PV, less common in HV and HA, and not observed at all in the CV and sinusoids. Similarly, CD200^+^ ECs were common in the PV, HV, and HA but not in the CV and sinusoids [2]. At E18, CD200 expression was confined to some PV ECs and increased after birth (Fig. 2g, Additional file 1: S2C). By P7, some HV ECs started expressing CD200, but the amount was lesser compared to PV ECs (Additional file 1: Fig. S2C and F). On the other hand, CD157 expression was hard to detect until around 3 weeks after birth, when the higher number of CD157^+^CD200^+^ ECs allowed easier identification. At P21, CD157 was found to be expressed mainly by ECs of the PV, less frequently in the HV, and was co-localized with CD200^+^ (Fig. 2g, Additional file 1: Fig. S2D and G). These data suggest that CD157^+^CD200^+^ VESCs emerged during late fetal and early neonatal development, possibly from a CD157^−^CD200^+^ EC population.

### Identification of VESC-like populations in perinatal micro- and macrovascular ECs

**Fig. 3:**
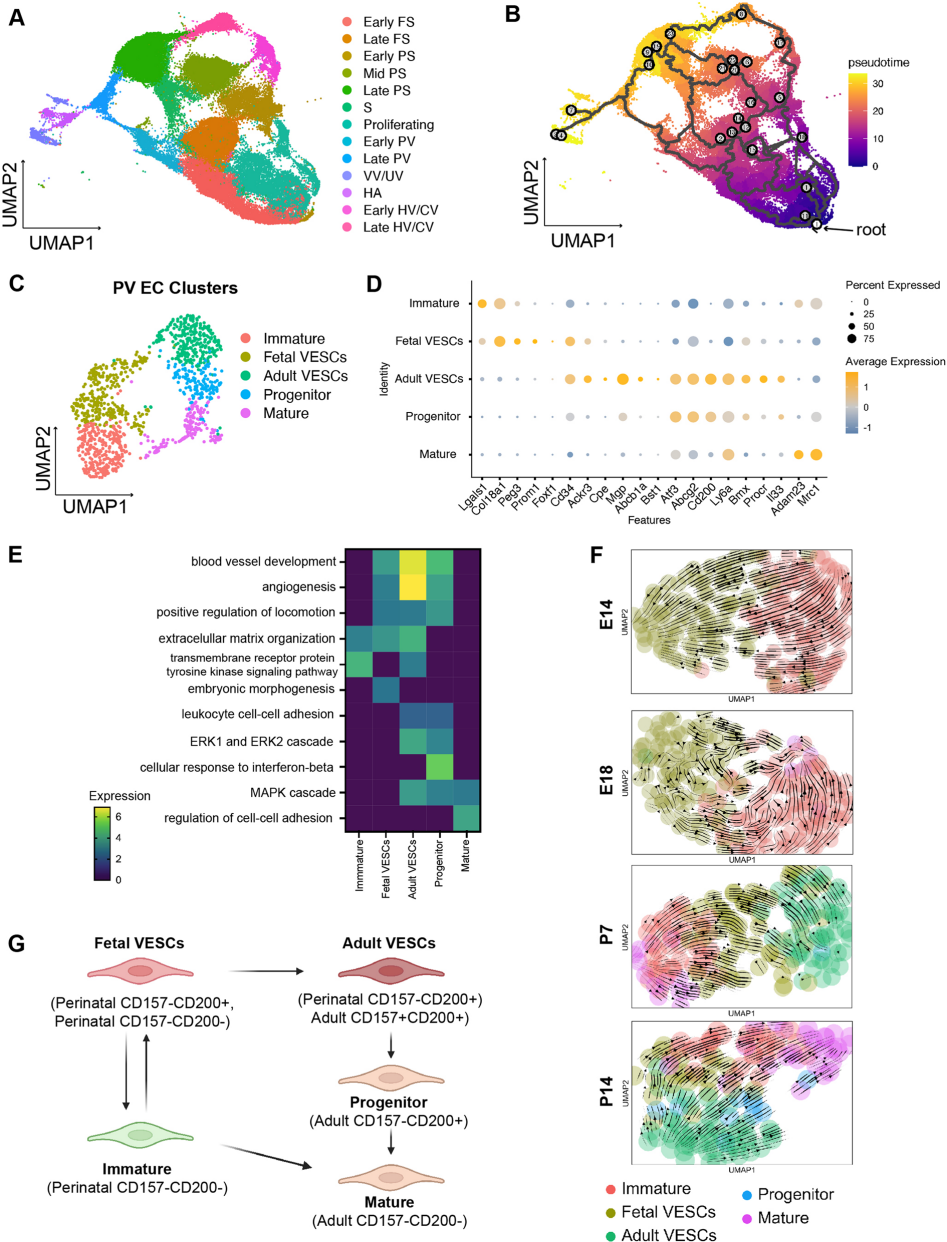
Trajectory analysis reveals stem cell-like populations in perinatal micro- and macrovascular ECs. **a** UMAP visualization of ECs from this study and published data [10]. **b** Trajectory analysis of ECs from E12 until W8 projected onto the UMAP plot. **c** UMAP embedding of PV EC clusters colored and labeled by cell type. **d** Dot plot of known endothelial stem cell or progenitor marker expression in PV EC clusters. **e** Comparison of GO biological process enrichment in each cluster. **f** RNA velocity from E14 through P14 illustrating development of fetal VESCs at E14 and transition from fetal VESCs to adult VESCs at P7. **g** Model of EC development in the portal vein and hepatic artery. Arrows indicate the possible differentiating and reprogramming directions

To investigate the development and differentiation of liver ECs and VESCs, trajectory analysis was performed using Monocle 3 [12–14]. By combining our EC data with the perinatal EC data from Gómez-Salinero et al. [10], we can visualize a complete view of EC development from E12 to W8 (Fig. 3a). Sinusoids from the early fetal period were defined as the root cells (Fig. 3b). Starting from the root, sinusoids matured from *CD34-*expressing early FS into late FS, early PS, mid-PS, and *Lyve1*+ midzonal late PS (Fig. 3b, Additional file 1: Fig. S3A and B). Early PV is likely differentiated from early FS, which further matures into late PV and gives rise to *Msr1*+ periportal late PS. On the other hand, HV/CV ECs developed during the late fetal or early postnatal period from early PS ECs, turned into late HV/CV ECs, and differentiated into *Kit*+ pericentral late PS. These results show that during the perinatal period, both PV and HV/CV ECs mainly develop from sinusoids but are then diverted away from the sinusoid trajectory, suggesting that macrovascular ECs possess their own stem cells. In contrast, trajectory mapping in late postnatal/adult periods revealed the emergence of sinusoids from PV and HV/CV ECs, possibly from adult CD157^+^CD200^+^ ECs (Additional file 1: S3B). VV/UV and HA EC clusters were located separately from the remaining clusters, which indicates that they follow a different developmental trajectory.

To explore adult CD157^+^CD200^+^ ECs fate specification, we performed re-clustering analysis on the PV EC cluster which has the highest number of CD157^+^CD200^+^ ECs. The analysis revealed five distinct clusters with segregation based on their differentiation potential (Fig. 3c). Immature clusters comprised mainly ECs from the perinatal period, which highly expressed *Lgals1*, an angiogenesis promoting gene [28] (Fig. 3c and d, Additional file 1: S3C). Two VESC-like cell clusters were identified based on the expression of *CD157/Bst1* and *CD200*: fetal and adult VESCs clusters (Fig. 3c, Additional file 1: S3D)*.* Both clusters shared the expression of *Cd34* [29]*, Cpe, and Ackr3* a G-protein-coupled receptor essential for vasculogenesis and angiogenesis [30], and other known endothelial stem cells/progenitor markers including *Peg3* [31], *Prom1/Cd133* [32]*, Foxf1* [33], *Abcb1a* [34]*, Abcg2* [2]*, Sca1/Ly6a* [35]*, Procr* [36]*,* and *Il33* [37] (Fig. 3c and d)*.* The progenitor cluster has similar but lower expression of adult VESC genes, i.e., *Bmx*, *Procr*, and others, reflecting a transition state toward mature ECs. The mature and the immature clusters were distinguished from the other clusters by expression of sinusoid markers such as *Adam23* and *Mrc1* (Fig. 3d). A total of 352 significant differentially expressed genes (DEGs) between clusters were identified, and gene ontology (GO) analysis revealed the highest enrichment of the genes involved in vasculature development, angiogenesis, and locomotion regulation in all VESCs and progenitor clusters, confirming a phenotype associated with stem/progenitor-like functions (Fig. 3e).

RNA velocity analysis confirmed the development of the fetal VESCs (CD157^−^CD200^+^ ECs) pool from immature ECs in the fetal period (Fig. 3f and g, Additional file: Fig. S3D). Such fetal VESCs could switch back to immature ECs, eventually transitioned into mature ECs postnatally. These immature ECs and fully transitioned mature ECs lost the ability to convert to fetal VESCs by P14. At P7, fetal VESCs started transitioning into adult VESCs (CD157^+^CD200^+^ ECs), which became the source of other ECs in adult PV. As the number of VESC-like cells expressing *CD200* increased in VESCs clusters, a *CD157/Bst1-*negative progenitor cluster emerged at P14. By P14, a clear differentiation from adult VESCs to mature ECs through progenitors was observable, which was previously described in adult liver ECs [2]. In addition, by ordering transcriptomes along a latent timeline, several putative driver genes, including *Myof* and *Wipf3,* were identified during the establishment of adult VESCs at P7 (Additional file 1: Fig. S3E and F). Recent studies highlight the importance of *Myof* for the proliferation and migration of ECs in response to vascular endothelial growth factor (VEGF), while *Wipf3* is involved in podosome formation in macrophages [38, 39]. However, the expression and function of *Wipf3* in ECs are obscure. Notably, *Myof* and *Wipf3* were also expressed by CD157^+^CD200^+^ ECs in HA and HV/CV EC clusters (Additional file 1: S3G and H). These findings imply that VESC-like cells reside within the microvascular CD157^−^CD200^−^ ECs (sinusoids) and macrovascular CD157^−^CD200^+^ ECs (VV/UV, PV, HA, and HV ECs) during the perinatal period, while in adults, VESC-like cells are mainly present in macrovascular CD157^+^CD200^+^ ECs (PV, HA, and HV ECs).

### Perinatal CD157^−^CD200^+^ ECs and adult CD157^+^CD200^+^ ECs possess similar stem cell potential

**Fig. 4:**
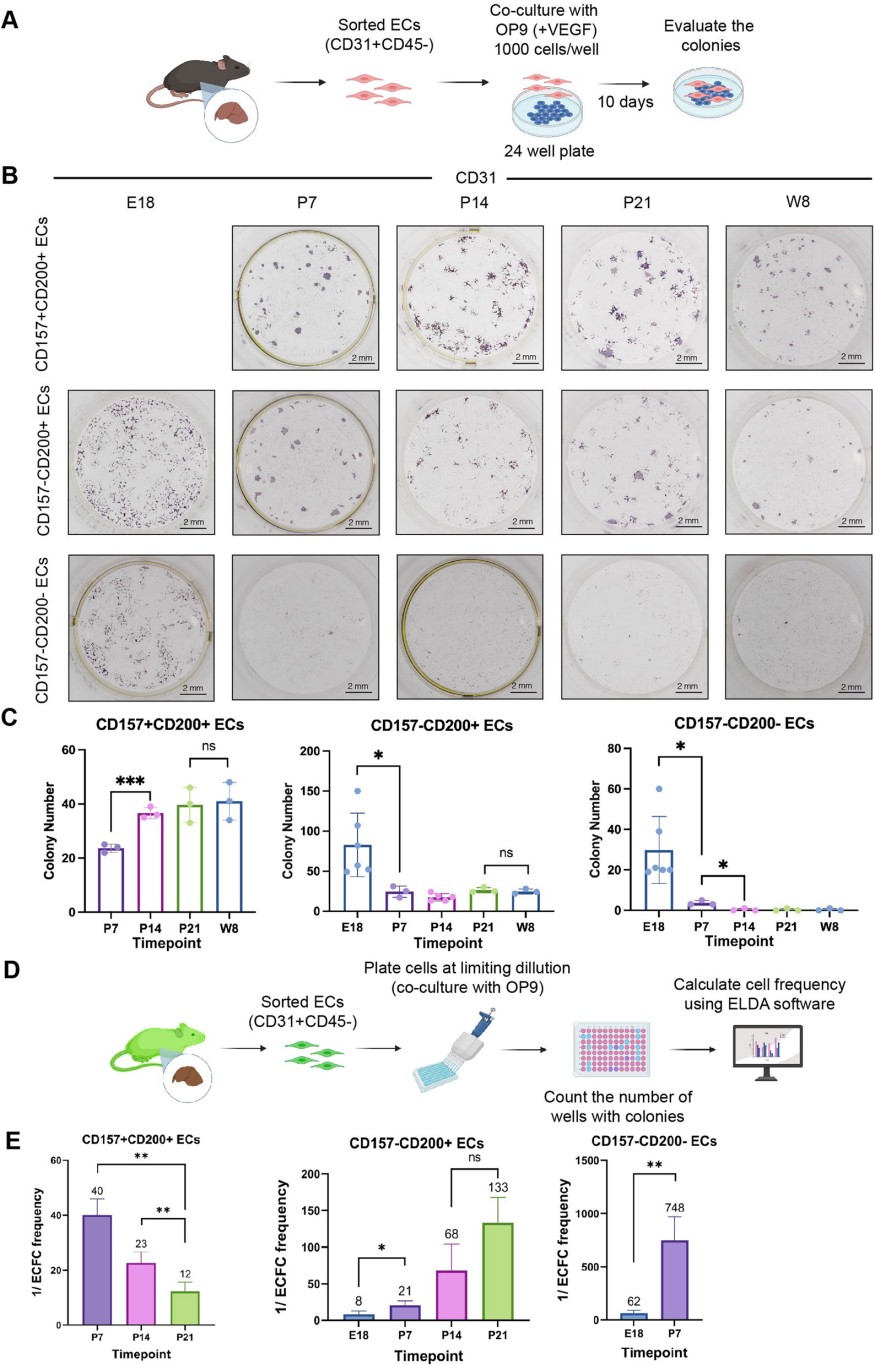
Perinatal CD157^−^CD200^+^ ECs and adult CD157^+^CD200^+^ ECs possess similar endothelial colony-forming ability. **a** Schematic overview. A total of 1000 liver ECs from fetal, neonatal, and adult liver were cultured on OP9 feeder cells for 10 days. **b **Cultured ECs from different time points were stained for CD31 (low-power view). The colony is defined as a cluster of > 50 ECs. There are no CD157 + CD200 + ECs at day E18. Experiments were repeated at least three times. **c** Quantification of the number of EC colonies formed by each fraction of ECs across development. **d** Schematic overview. Limiting dilution analysis of liver ECs from GFP mice cultured on OP9 feeder cells for 10 days. Experiments were repeated at least three times. **e** ECFCs frequency in each EC fraction calculated using the online algorithm, n = 3. Data are shown as means ± SEM. Statistical analysis using unpaired two-tailed t-test, ***p < 0.0001, **p < 0.005, and *p < 0.05, ns *p* > 0.05

We previously used co-cultures of ECs with OP9 stromal layers to demonstrate the high proliferative ability of VESCs (CD157^+^CD200^+^ ECs) [2]. In the present study, we utilized the same co-culture system to test the ability of ECs from animals in the perinatal period to form endothelial colonies. Ten days after culture initiation, the colonies were enumerated by staining cells in the culture plates with anti-CD31 antibody. A colony was defined as a cell cluster composed of > 50 ECs [40] (Fig. 4a). As expected, the colony-forming ability is restricted almost exclusively to CD157^+^CD200^+^ ECs or the CD157^−^CD200^+^ ECs (Fig. 4b). Surprisingly, CD157^−^CD200^+^ ECs from E18 had the greatest colony-forming ability. CD157^−^CD200^−^ ECs could also generate endothelial colonies, albeit fewer than CD157^−^CD200^+^ ECs from animals at the same developmental stage. However, the size of the colonies derived from fetal ECs in either the CD157^−^CD200^−^ or CD157^−^CD200^+^ EC fraction was smaller than of adult CD157^+^CD200^+^ ECs (Fig. 4c, Additional file 1: Fig. S4A). As mentioned before, the CD157^+^CD200^+^ ECs population emerged around the 1st week after birth. At P7, the colony-forming ability of CD157^+^CD200^+^ ECs and CD157^−^CD200^+^ ECs was similar but decreased in CD157^−^CD200^−^ ECs. However, as the number of CD157^+^CD200^+^ ECs increased, the colony-forming ability of CD157^−^CD200^+^ ECs decreased (Fig. 4c). We considered the possibility that OP9 stromal cells affect the ability of certain ECs to generate colonies. To exclude this, we cultured ECs from P14 in laminin-coated dishes and found that the efficiency of their acquiring a cobblestone appearance was equivalent to the colony-forming ability of these ECs in the absence of OP9 cells (Additional file 1: Fig. S4B).

To quantify the frequency of cells capable more precisely of producing colonies in each fraction, we sorted ECs from C57BL/6-Tg (CAG-EGFP) mice and plated them at limiting dilutions. After 10 days, we counted the number of wells with colonies and calculated the frequency of endothelial colony-forming cells (ECFCs) using ELDA software [7] (Fig. 4d). Confirming colony-forming analysis results as above, in the CD157^+^CD200^+^ EC fraction, the frequency of cells which possessed colony-forming capacity increased with age, indicating that ECs in this population become more homogeneous in terms of the stem cell-containing fraction. At P21, it was estimated that one in 12 CD157^+^CD200^+^ ECs fraction possessed colony-forming ability, and this number is expected to increase until adulthood (Fig. 4e). In contrast, the frequency of colony-forming cells within the CD157^−^CD200^+^ ECs population decreased with age (one in eight cells at E18 but only one in 133 at P21). This suggests the enriched CD200 expression in highly proliferating ECs in late fetal development but then the CD157^−^CD200^+^ ECs fraction became more heterogeneous with the emergence of CD157^+^CD200^+^ ECs. Meanwhile, in the CD157^−^CD200^−^ ECs fraction, cells with the capacity to form colonies were found only in the late fetal (one in 62 cells) and early neonatal period (one in 748 cells) (Fig. 4e).

**Fig. 5:**
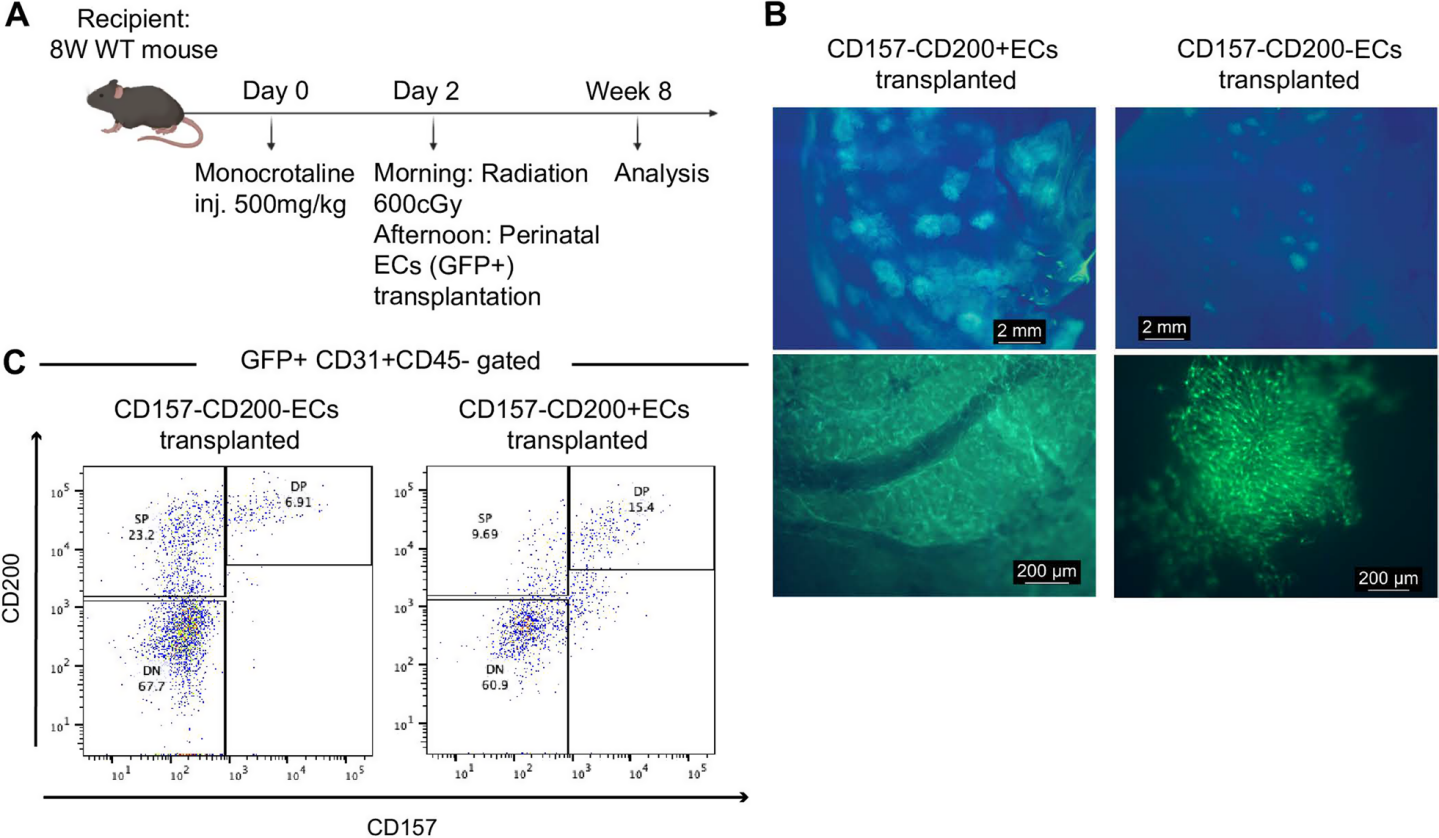
Perinatal CD157^−^CD200^−^ ECs and CD157^−^CD200^+^ ECs generate CD157^+^CD200^+^ ECs in vivo. **a** Schematic depicting the EC transplantation workflow. Experiments were repeated at least three times. **b** Representative images of recipient liver transplanted with perinatal CD157^−^CD200^−^ ECs and CD157^−^CD200^+^ ECs after 2 months. **c** FACS analysis of the recipient mouse liver showing the development of CD157^+^CD200^+^ ECs from perinatal CD157^−^CD200^−^ ECs and CD157^−^CD200^+^ ECs

Finally, to confirm the development of CD157^+^CD200^+^ ECs in vivo, perinatal CD157^−^CD200^−^ ECs and CD157^−^CD200^+^ ECs from E18 CAG-EGFP mouse liver were transplanted into recipient adult wild-type mice after liver injury (Fig. 5a). After 8 weeks, we observed that the GFP + cells from both fractions were incorporated into the liver vasculature (Fig. 5b). Unlike adult CD157^−^CD200^−^ ECs which only gave rise to CD157^−^CD200^−^ ECs [2], perinatal CD157^−^CD200^−^ ECs were able to generate all EC fractions, confirming the contribution of sinusoid to macrovascular EC development (Fig. 5c). Similarly, adult CD157^−^CD200^+^ ECs did not give rise to CD157^+^CD200^+^ ECs but perinatal CD157^−^CD200^+^ ECs were able to do so, indicating that ECs marked as CD157^−^CD200^+^ contain cells transitioning from fetal to adult VESC-like cells.

## Discussion

The existence of stem and progenitor ECs in the perinatal period and the developmental origin of previously identified resident VESCs has remained unclear thus far. Through scRNA-seq analysis, we documented the heterogeneity of macrovascular ECs and the existence of fetal and adult VESC-like cell populations within PV ECs, which have self-renewal properties and give rise to differentiated PV ECs and periportal sinusoids. Similarly, trajectory analysis also showed the emergence of pericentral sinusoids from HV ECs. This finding had been reported before in adult liver as CD157^+^CD200^+^ VESCs express *Bmx* and lineage tracing using Bmx(PAC)-CreERT2/Flox-CAT-EGFP mice during steady-state conditions revealed that EGFP + VESCs replace both vascular ECs and sinusoids in the long term [2]. Experiments on adult CCl4-induced liver injury also revealed the emergence of new sinusoid expressing *CD34* and *Ednrb* which are normally only expressed by PV EC clusters, suggesting that sinusoid developed from PV ECs [41].

In the fetal liver, VESC-like cells exclusively express *CD200*, but in the postnatal liver, *CD200* is expressed on both VESCs and progenitor-like cells. On the other hand, *CD157* was exclusively expressed by VESC-like cells. Using transplantation assay, we identified the emergence of adult CD157^+^CD200^+^ VESCs from perinatal CD157^−^CD200^+^ VESC-like cells. We demonstrated that this EC population possessed a high proportion of ECFCs with similar colony-forming ability to adult CD157^+^CD200 + VESCs. This perinatal CD157^−^CD200^+^ or fetal VESC-like cells highly express known markers of stem or progenitor populations such as *Cd34, Peg3*, *Prom1,* and *Foxf1*. Of these markers, *Peg3* is highly enriched in Fetal VESCs cluster and downregulated in adult VESCs cluster. A previous study showed that the fetal Peg3^+^ ECs were highly proliferative and readily able to form new blood vessels in vivo [31]. Our study also confirms the previous findings on the presence of endothelial progenitors in fetal liver which were described as CD31^+^Sca1^+^ ECs able to form new vessels in vitro and in vivo [42]. Developmental switching from fetal to postnatal phenotype VESC-like cells occurs around 1 week after birth. We identified several putative genes as drivers of this commitment, such as *Myof* and *Wipf3*. However, future studies are needed to clarify the function of these genes for the development of adult VESC-like cells. In addition, we also identified hierarchical trajectories of development from VESC-like cells to mature ECs through progenitor-like cells beginning in P14 liver [43].

Trajectory analysis revealed that perinatal CD157^−^CD200^+^ ECs were derived from a progenitor population within sinusoid ECs. This population expresses *Cd34,* which is consistent with the previous studies identifying a sinusoid progenitor (CD45^−^FLK1^+^CD31^+^CD34^+^ ECs) in E12 liver [10, 44]. However, the progenitor activity of this population is restricted in fetal and early postnatal periods because adult sinusoids no longer express *Cd34*. Another study also revealed that the gene expression of iPS-derived vascular progenitor ECs (vECs) is more similar to fetal than to adult sinusoids [45]. Consistent with our findings, in early human fetal liver, ECs are mainly derived from CD34^+^LYVE1^+^ sinusoid ECs [46]. During intermediate fetal development, these cells differentiated into PV ECs prior to CV ECs, marked by early downregulation of sinusoid marker LYVE1 [10].

The existence of multiple stem cell populations in ECs is not a novel finding. In early liver morphogenesis, PV, HV, and sinusoids have different origins. Based on recent evidence, it has been proposed that the vitelline vein gives rise to the portal vein; the posterior cardinal veins give rise to the hepatic vein and the central vein, and both the vitelline and the posterior cardinal veins give rise to the liver sinusoids [47]. Another study revealed, sinusoids can be derived from other sources including sinus venous-derived NFATC1^+^ and NPR3^−^ endothelial progenitors, hemangioblasts, and erythro-myeloid progenitors [48].

Our study has some limitations. Progenitor/stem cell-like populations identified in this study might be specific only for C57BL/6 mouse liver ECs, because EC heterogeneity may exist between tissues, strains, and species. The previous studies compared transcriptomic properties between C57BL/6 and SCID mouse liver ECs, showing strain-specific gene expression and suggesting that immune status contributes to the transcriptional profile [49].

## Conclusion

In this study, we conducted a comprehensive atlas of liver EC populations in mice, offering insight into micro- and macrovascular ECs heterogeneity and its development potential. Our data add another layer of evidence regarding the existence of VESC-like cells across development and illustrate a hierarchical development from VESCs to progenitor cells to mature ECs. Future studies will focus on clarifying the molecular determinant of adult VESCs development.

### Supplementary Information

**Additional file 1: Figure S1.** Preliminary analysis and comparison of scRNA-seq data.** Figure S2.** FACS and IF staining of CD157 and CD200 in the liver from the perinatal period.** Figure S3.** Dynamic changes in gene expression during adult VESCs specification.** Figure S4.** Colony-forming ability of EC fractions from perinatal liver.

## Data Availability

The scRNA-seq raw data are deposited in the NCBI Gene Expression Omnibus (GEO) database under accessions number GSE231545 (publicly available at https://www.ncbi.nlm.nih.gov/geo/query/acc.cgi?acc=GSE231545) and available from the corresponding author on reasonable request.

## Abbreviations

VESC: Vascular endothelial stem cell
ECs: Endothelial cells
E: Embryonic
P: Postnatal
W: Week
EPCs: Endothelial progenitor cells
STM: Septum transversum mesenchyme
HSCs: Hematopoietic stem cells
scRNA-seq: Single-cell RNA sequencing
FBS: Fetal bovine serum
VEGF: Vascular endothelial growth factor
FACS: Fluorescence-activated cell sorting
UMAP: Uniform manifold approximation and projection
GO: Gene ontology
MCT: Monocrotaline
IF: Immunofluorescence
FS: Fetal sinusoids
PS: Postnatal sinusoid
S: Sinusoids
PV: Portal vein
HV: Hepatic vein
CV: Central vein
VV: Vitelline vein
UV: Umbilical vein
HA: Hepatic artery
SEM: Standard error of the mean
ECFCs: Endothelial colony-forming cells
