# Supplementary material for: Single-cell sequencing reveals the existence of fetal vascular endothelial stem cell-like cells in mouse liver
Source: Stem Cell Res Ther. 2023 Aug 30;14:227. doi: 10.1186/s13287-023-03460-y (PMC10468894; doi:10.1186/s13287-023-03460-y)
Supplement: Supplementary file 1 — Additional file 1: Figure S1. Preliminary analysis and comparison of scRNA-seq data. Figure S2. FACS and IF staining of CD157 and CD200 in the liver from the perinatal period. Figure S3. Dynamic changes in gene expression during adult VESCs specification. Figure S4. Colony-forming ability of EC fractions from perinatal liver. [file 13287_2023_3460_MOESM1_ESM.docx]

**
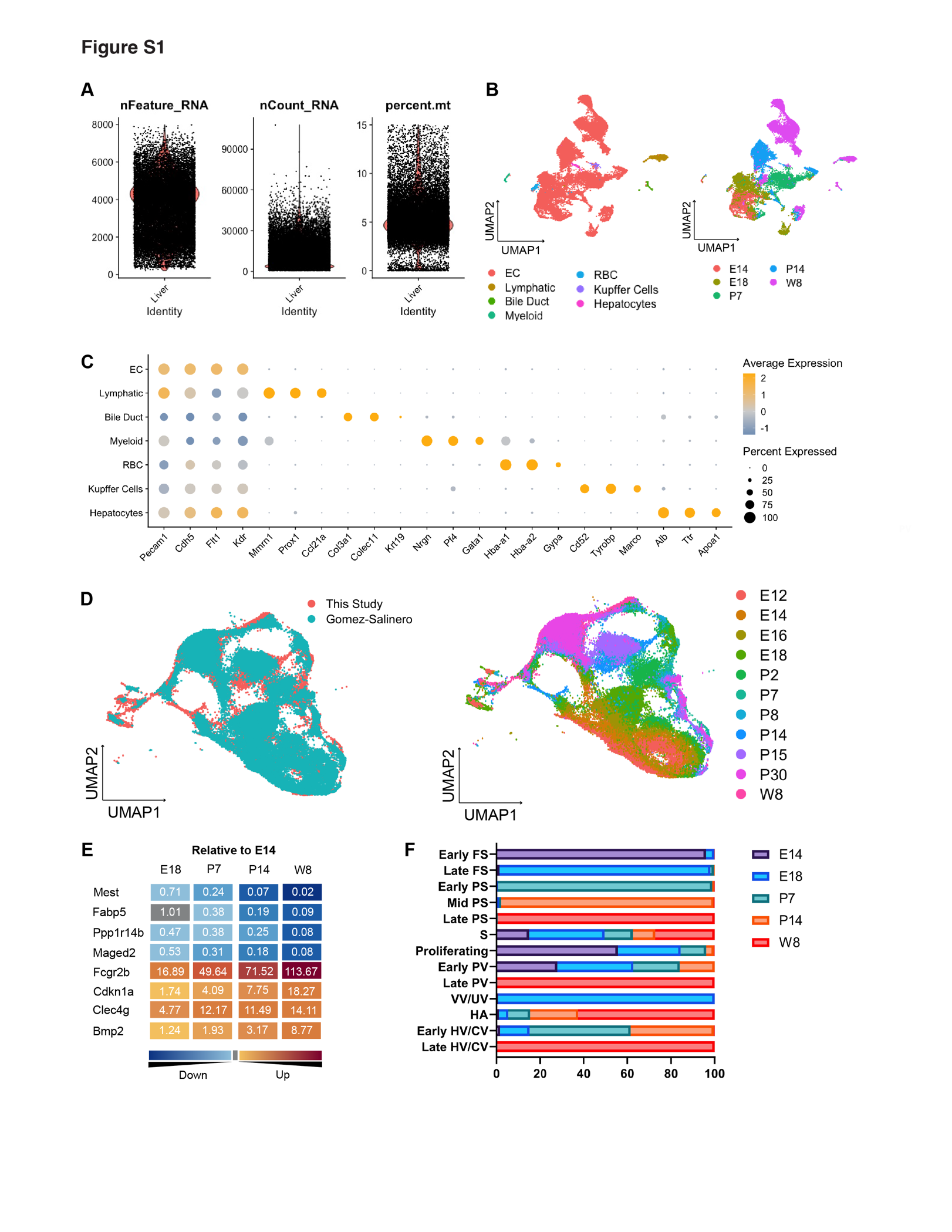
**

**Figure S1** Preliminary analysis and comparison of scRNA-seq data. **A.** Quality control for scRNA-seq data. Cells with <200 genes, >8000 genes, or >15% mitochondrial counts were filtered out. **B.** UMAP plot of all cells from E14, E18, P7, P14, and W8 (n=22.385). Colored and labelled by cell type (left) and timepoint (right). **C.** Dot plot depicting differentially expressed genes for all cell types identified. **D.** UMAP plots of integrated data from this study and Gómez-Salinero et al. Colored and labeled by data source (left) and timepoint (right). **E.** Fold-changes of sinusoids gene expression at each timepoint relative to E14. Red indicates upregulation, blue indicates downregulation. **F.** Changes of proportions in each EC cluster from fetal to adult.

**
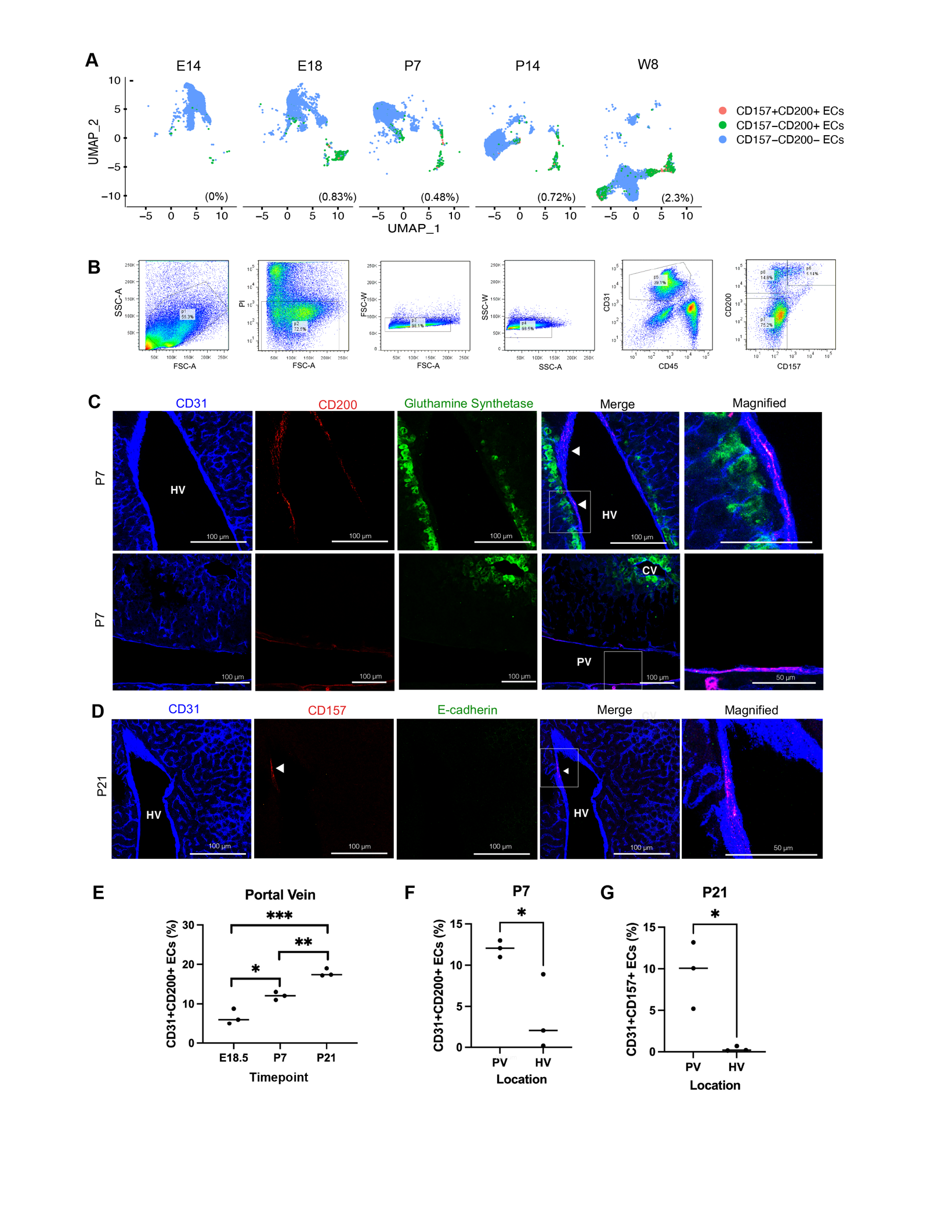
**

**Figure S2** FACS and IF staining of CD157 and CD200 in the liver from the perinatal period. **A**. UMAP visualization of PV ECs based on both *CD157* and *CD200* expression for each timepoint. **B.** FACS gating strategy for analyzing CD157 and CD200 expression on liver ECs. Total cells were first gated according to forward scatter (FSC) and side scatter (SSC), followed by exclusion of dead cells and doublets. These cells were further gated for the EC subset, CD31^+^CD45^-^ before analyzing CD157 and CD200 expression. **C.** The expression of CD200 (white arrow heads) was detected in hepatic vein but not in central vein at P7. HV: hepatic vein; PV: portal vein; CV: central vein **D.** CD157 expression (white arrow heads) was detected in the hepatic vein of P21 liver. **E and F.** Quantification of CD31^+^CD200^+^ ECs by immunofluorescence staining based on timepoint **(E)** and location **(F)**. **G.** Quantification of CD31^+^CD157^+^ ECs by immunofluorescence staining based on location. Statistical analysis using unpaired two-tailed t test, ^∗∗∗^p < 0.0001, ^∗∗^p < 0.005, ^∗^p < 0.05.

**
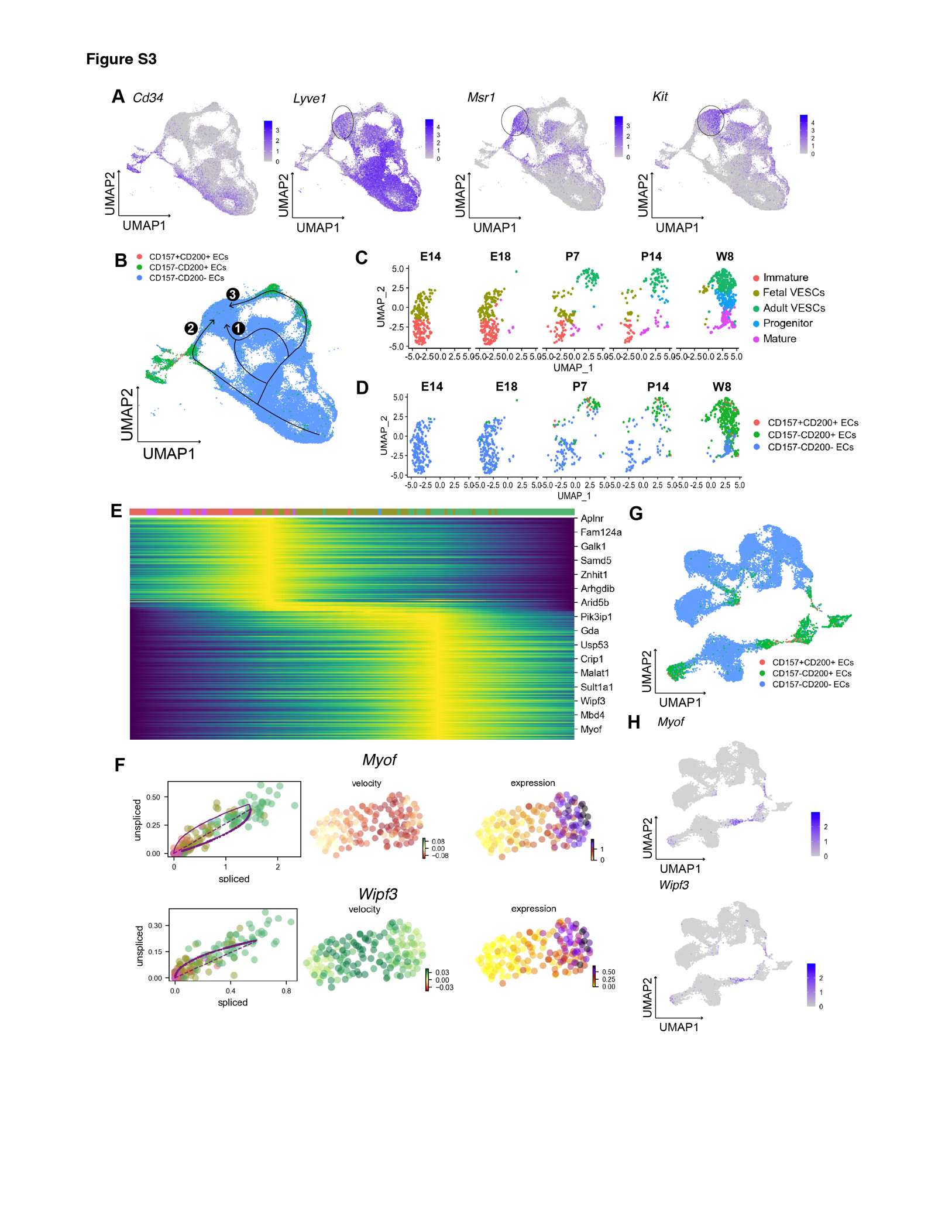
**

**Figure S3** Dynamic changes in gene expression during adult VESCs specification. **A.** Feature plots showing expression of *Cd34, Lyve1, Msr1* and *Kit*. Each purple dot represents a single cell. **B.** UMAP plot showing the direction of differentiation based on *CD157* and *CD200* expression. Arrow 1: sinusoid differentiation; Arrow 2: PV differentiation; Arrow 3: HV/CV differentiation. **C.** UMAP plots of PV EC clusters stratified by developmental stages. **D.** UMAP visualization of PV ECs based on *CD157* and *CD200* expression. **E.** Heatmap of the top 300 likelihood-ranked genes along latent time. **F.** Phase portraits, RNA velocity, and expression dynamic along latent time of putative driver genes. **G.** UMAP visualization of ECs based on *CD157* and *CD200* expression. **H.** Expression of *Myof* and *Wipf3* in CD157^+^CD200^+^ ECs.

**
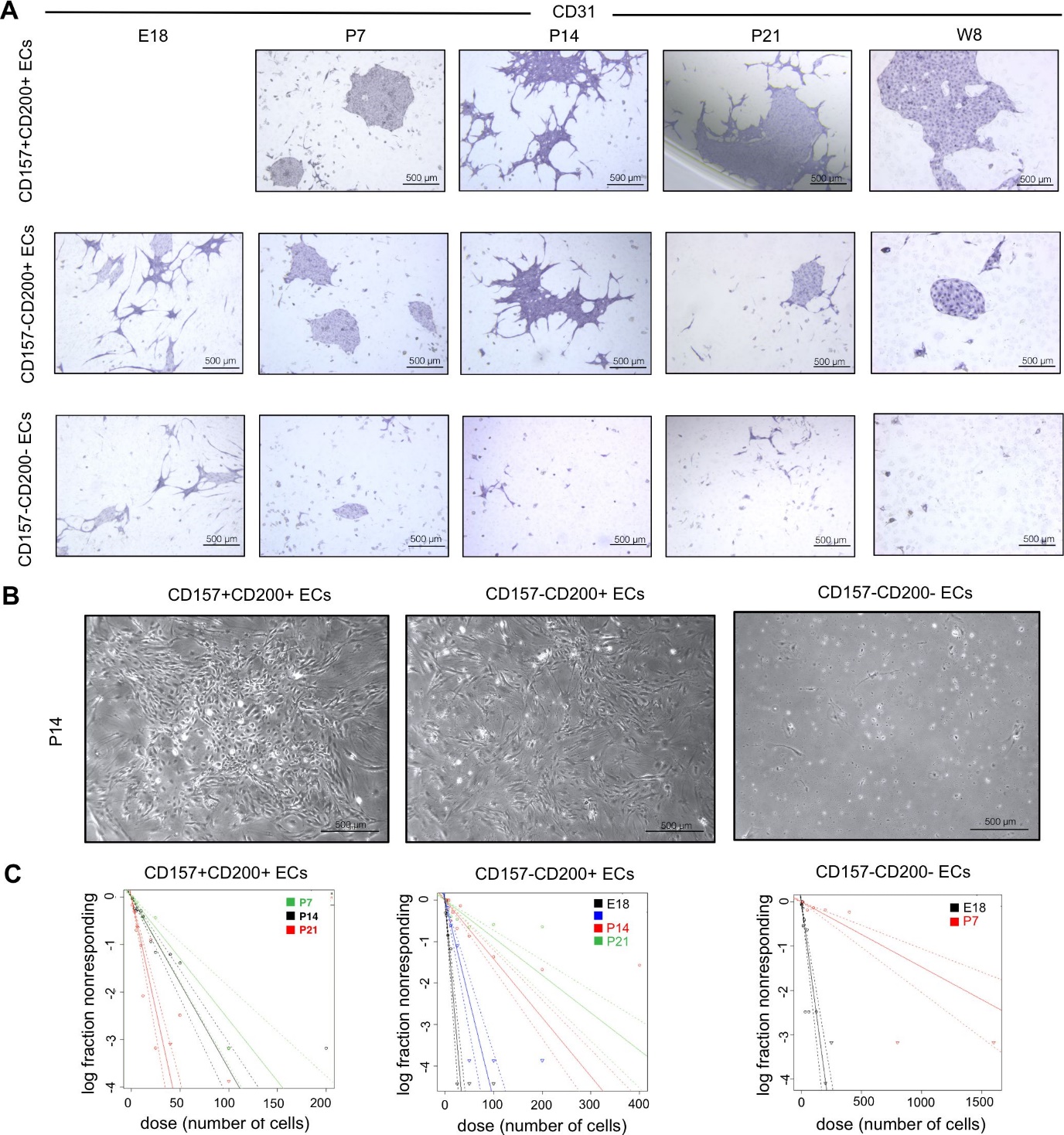
**

**Figure S4** Colony-forming ability of EC fractions from perinatal liver. **A.** ECs cultured on OP9 were stained for CD31 (high-power view). There is no CD157^+^CD200^+^ ECs at day E18. **B.** 10.000 ECs from P14 were cultured on laminin-511-coated dishes. ECs from CD157^+^CD200^+^ fraction showed characteristic cobblestone morphology. **C.** Graphs show the number of seeded cells (*x*-axis) plotted against the log fraction of wells without any colonies (*y*-axis). The slope of the line represents the log-active cell fraction. Left panel: CD157^+^CD200^+^ ECs; ECs from P7, P14, P21 are depicted in green, black and red, respectively. Middle panel: CD157^-^CD200^+^ ECs; ECs from E18, P7, P14, P21 are depicted in black, blue, red, and green, respectively. Right panel: CD157^-^CD200^-^ ECs; ECs from E18 and P7 are depicted in black and red, respectively.
